# A Pseudo-Right Atrial Mass in Massive Pulmonary Embolism: A Case Report Highlighting Multimodality Imaging and Multidisciplinary Team Review

**DOI:** 10.7759/cureus.98329

**Published:** 2025-12-02

**Authors:** Mehak Gupta, Aaditya Kodamanchile, Louise Tamin, Pavithralakshmi Venkatraghavan, Andrew Cole

**Affiliations:** 1 Cardiology, University Hospitals of North Midlands NHS Trust, Stoke-on-Trent, GBR; 2 Medicine, Hereford County Hospital, Hereford, GBR

**Keywords:** direct oral anticoagulant therapy, mri cardiac, multidisciplinary teams, right atrial cardiac mass, transthoracic echocardiogram

## Abstract

Transthoracic echocardiography (TTE) is often the first-line imaging modality in cardiac assessment due to its accessibility and rapid acquisition. However, compared with cardiac magnetic resonance (CMR), it offers limited tissue characterisation and may misinterpret anatomical variants as pathology. Consequently, multimodality imaging and multidisciplinary team (MDT) review are frequently required for accurate diagnosis. A 44-year-old man presented with acute dyspnoea following long-haul travel. Computerised tomography pulmonary angiography revealed massive bilateral pulmonary emboli without right ventricular strain, and he was treated with rivaroxaban 20 mg once a day. Three months later, follow-up TTE demonstrated a right atrial mass, reported as a possible thrombus, prompting anticoagulation change to warfarin. At six months, CMR showed no intracardiac mass. However, repeat TTE at one year again suggested a right atrial mass. A review during the cardiac imaging MDT concluded that the apparent “mass” represented epicardial fat entering the imaging plane. Rivaroxaban 20 mg once a day was reinstated for indefinite anticoagulation. This diagnostic pitfall led to an unnecessary switch to warfarin, highlighting how misinterpretation of anatomical variants can significantly alter clinical management. This case underscores the critical role of multimodality imaging and MDT review in the evaluation of intracardiac masses to prevent misdiagnosis and unnecessary treatment.

## Introduction

Intracardiac masses encompass a broad differential, including thrombi, benign and malignant tumours, and infective vegetations. Their diagnosis can be challenging, as both clinical presentation and imaging appearances may be non-specific [1]. Transthoracic echocardiography (TTE) remains the initial diagnostic tool of choice, but it has limitations in tissue characterisation and in differentiating normal anatomical variants from true pathology. Cardiac magnetic resonance (CMR) provides superior spatial resolution and tissue characterisation, making it invaluable when echocardiographic findings are equivocal [2]. Multimodality imaging combined with multidisciplinary team (MDT) interpretation is recognised as essential to achieve diagnostic accuracy and guide management. Epicardial fat is a frequent mimic of intracardiac masses and can often be misinterpreted. Correct interpretation is crucial, as misdiagnosis can lead to unnecessary therapies, invasive investigations, or inappropriate long-term treatment.

Here, we present the case of a gentleman who had an incorrectly diagnosed atrial thrombus from TTE, leading to a change in anticoagulation therapy.

## Case presentation

A 44-year-old man presented with a four-day history of progressive dyspnoea following a recent long-haul flight. His medical history included recurrent iron deficiency anaemia, a provoked deep vein thrombosis in 2015, and a confirmed prothrombin G20210A mutation. In childhood, he underwent repair of a tracheo-oesophageal fistula with left partial lung lobectomy for empyema. He was otherwise healthy, a regular runner, and a lifelong non-smoker. On examination, he was haemodynamically stable (blood pressure, 157/90 mmHg; heart rate, 95 beats/minute; oxygen saturation, 95% on room air) with unremarkable cardiorespiratory findings. Assessment of pulmonary embolism severity indicated a very low risk (Pulmonary Embolism Severity Index score 54). Laboratory investigations revealed a normal full blood count and N-terminal pro-B-type natriuretic peptide, with an elevated D-dimer of 13,314 µg/L (Table 1).

**Table 1:** Biochemical results from the initial admission. NT-proBNP: N-terminal pro-B-type natriuretic peptide

Blood test	Result	Normal range
Haemoglobin	164	130–180 g/L
White cell count	12.2	4–11 × 10^9^/L
Platelet count	291	150–450 × 10^9^/L
Haematocrit	0.492	0.37–0.51
Neutrophil count	9	2–7.5 × 10^9^/L
Lymphocyte count	2	1.5–4 × 10^9^/L
Sodium	139	133–146 mmol/L
Potassium	4.3	3.5–5.3 mmol/L
Urea	5.1	2.5–7.8 mmol/L
Creatinine	73	59–104 µmol/L
Glomerular filtration rate	>90	mL/minute/1.7m²
D-dimer	13,314	0–500 ng/mL
NT-proBNP	106	0–399 ng/L

**Figure 1:**
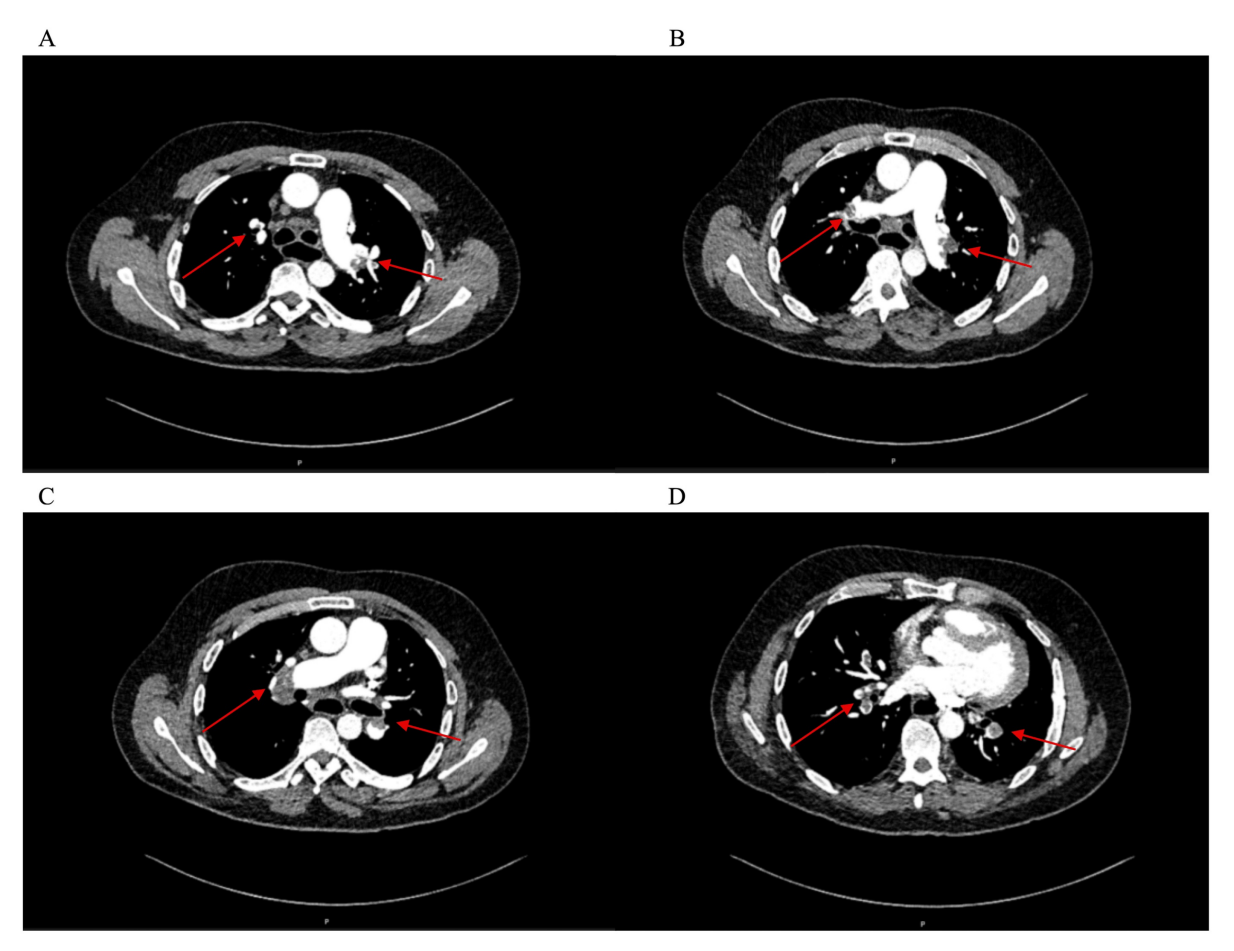
Axial contrast-enhanced CT pulmonary angiography demonstrating extensive bilateral pulmonary emboli. (A–D) Red arrows indicate intraluminal filling defects consistent with acute pulmonary emboli involving the segmental and subsegmental branches of the pulmonary arteries. Images illustrate emboli in both the right and left pulmonary arterial trees at different axial levels, showing extensive clot burden and distribution.

He underwent a CT pulmonary angiography, which demonstrated extensive bilateral pulmonary emboli involving the distal main, lobar, segmental, and subsegmental branches, with a pulmonary trunk diameter of 35 mm (Figure 1). A wedge-shaped opacity in the left lower lobe suggested early pulmonary infarction, and multiple centrilobular ground-glass nodules raised the possibility of an infective or inflammatory process. No right ventricular strain was seen. The patient was initiated on rivaroxaban 20 mg once daily and discharged the following day.

**Figure 2:**
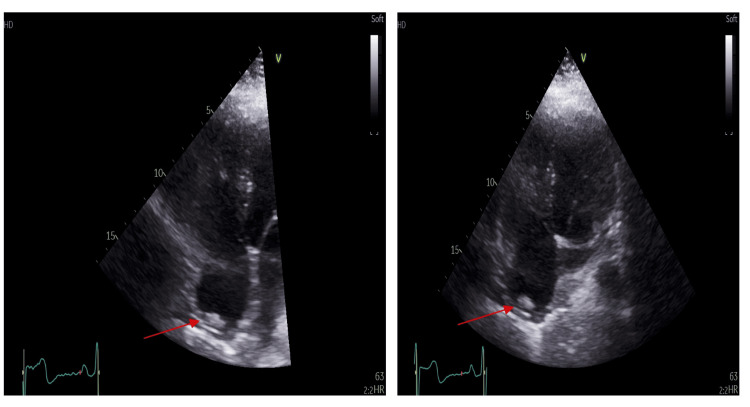
Transthoracic echocardiography demonstrating a right atrial mass. Apical four-chamber view showing a well-defined echogenic mass within the right atrium (red arrow).

**Figure 3:**
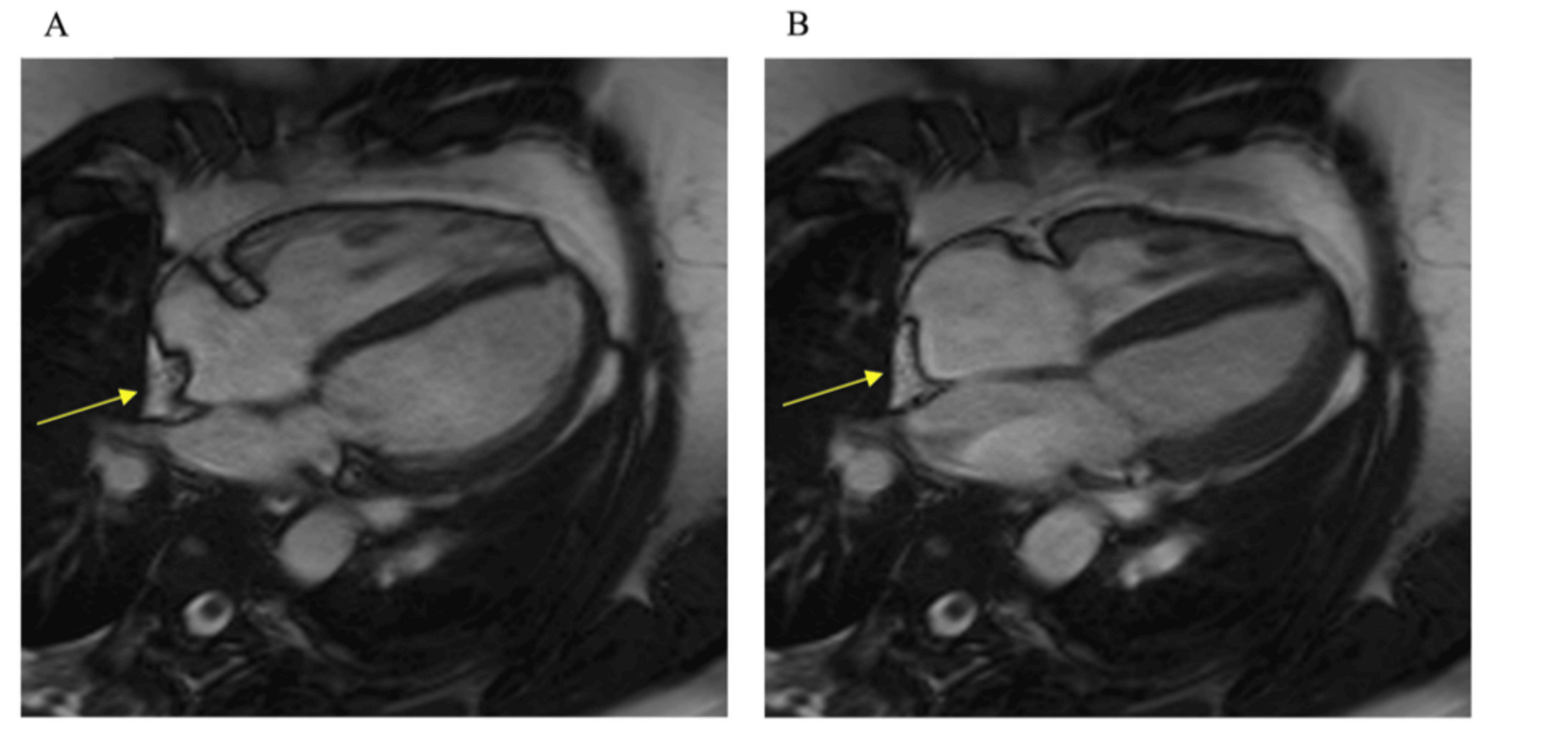
Cardiac magnetic resonance (CMR) imaging confirming absence of a true intracardiac mass. (A) Steady-state, free precession, four-chamber, cine CMR image showing extracardiac fat invaginating into the right atrium (yellow arrow). (B) End-systolic frame of the same image series with extracardiac fat (yellow arrow).

At the three-month follow-up, the patient underwent TTE, which showed a normal left ventricular cavity size and function (left ventricular ejection fraction (LVEF) >55%). His right ventricle was of normal size and function (tricuspid annular plane systolic excursion (TAPSE) = 20 mm, right ventricle S' = 14 cm/s). His right ventricular/left ventricular ratio was 0.75. There was no significant tricuspid regurgitation with no measurable Doppler trace. There was an echogenic mass in the right atrium, which was reported as a likely atrial thrombus (Figure 2). Based on this, anticoagulation was changed from rivaroxaban to warfarin under haematology guidance. At six months, CMR demonstrated normal left ventricular size and function (LVEF = 56%). His right ventricle was of normal size with normal function (TAPSE = 24 mm). There was no evidence of a right atrial mass, though an extracardiac origin of the previous echocardiographic finding could not be definitively excluded (Figure 3).

At one year, repeat TTE once again demonstrated the apparent right atrial mass. The case was discussed in the cardiac imaging MDT meeting, wherein both echocardiograms and the MRI were re-reviewed and compared. Comparative analysis of sequential TTEs and the prior CMR concluded that the echocardiographic appearances were due to epicardial fat protruding into the imaging plane rather than a true intracardiac mass. Anticoagulation with rivaroxaban 20 mg once daily was reinstated for indefinite therapy justified by the cumulative risk of the confirmed prothrombin G20210A mutation, recurrent venous thromboembolism history, and the massive burden of the current pulmonary embolism. The patient remains clinically well and continues regular follow-up with haematology and respiratory services (Table 2).

**Table 2:** Timeline of events. ED: emergency department; PE: pulmonary embolus; TTE: transthoracic echocardiogram; CMRI: cardiac magnetic resonance imaging; MDT: multidisciplinary team

Date/Interval	Event
Childhood	Repair of a tracheo-oesophageal fistula; partial lung resection
2015	Deep vein thombosis following knee injury; prothrombin gene mutation confirmed
2 weeks prior	Long-distance flight
Day –4	Onset of dyspnoea
Day 0	Presentation to ED; CT pulmonary angiography confirms massive bilateral PE
3 months	A suspicious right atrial mass detected on TTE
5 months	Anticoagulation switched from rivaroxaban to warfarin
6 months	No right atrial mass seen on CMRI
1 year	Repeat TTE shows an apparent right atrial mass
1 year + MDT review	Mass appearances on both echocardiograms agreed to represent epicardial fat
15 months	Rivaroxaban 20 mg once daily reinitiated

## Discussion

This case underscores the critical role of multimodality imaging in the assessment of suspected intracardiac masses and highlights the risks of over-reliance on a single imaging technique.

Cardiac masses represent a broad spectrum of neoplastic and non-neoplastic (pseudo-masses) lesions. Neoplastic masses include both primary and metastatic tumours, while pseudo-masses can include thrombi, vegetations, and calcific deposits [3]. Although most cardiac masses are benign, they can still cause morbidity through obstruction, infiltration, embolisation, arrhythmia, or even sudden death, making timely and accurate diagnosis essential [4]. True primary cardiac tumours are rare, with a prevalence of 0.15% in echocardiographic studies [5], whereas pseudo-masses are far more common in clinical practice [6].

TTE remains the first-line modality for the detection and initial assessment of cardiac masses due to its accessibility, portability, and ability to provide real-time haemodynamic assessment. Advanced echocardiography, such as three-dimensional, transoesophageal, and contrast-enhanced, offers additional detail regarding size, mobility, and attachment [7]. While TTE can have a sensitivity of around 90% for detecting cardiac masses, specificity can be quite variable, ranging from 50% to 90%. Use of contrast perfusion can improve both sensitivity and specificity to the mid-90% range [8]. However, its limited tissue characterisation and spatial resolution often necessitate further assessment with cardiac CT or CMR [9].

CMR is considered the gold standard for non-invasive tissue characterisation of cardiac masses, providing excellent soft-tissue contrast to distinguish fat, fluid, fibrosis, thrombus, and tumour. Importantly, CMR can prevent unnecessary surgery by confirming benign or thrombotic lesions. Limitations include lower temporal resolution, contraindications in patients with non-MRI-compatible devices, and difficulty evaluating small (<5 mm) or highly mobile lesions [10]. With optimal image quality, CMR achieves sensitivity and specificity near 90% for distinguishing benign from malignant lesions [11].

Epicardial fat is a frequent mimic of right atrial masses that is often misinterpreted as pathology, particularly in apical or subcostal views [12,13]. This is because the echogenic appearance can be similar to soft-tissue masses, and TTE often lacks the spatial resolution to clearly differentiate epicardial fat, the pericardium, and the right atrial wall, leading to misdiagnosis. Misinterpretation can lead to inappropriate management, as seen in this case, where prominent epicardial fat was mistaken for thrombus due to its echogenicity and location. This led to an unnecessary switch to warfarin, despite the patient’s prothrombin mutation effectively requiring stable long-term anticoagulation rather than variable vitamin K antagonist therapy.

In this patient, the suspicion of a right atrial mass prompted a change in anticoagulation and consideration of invasive diagnostics. Subsequent CMR excluded a true intracardiac lesion, identifying epicardial fat on fat-suppressed sequences [14]. Ultimately, the correct diagnosis was established only after an MDT review, comparing sequential echocardiograms with CMR images. This collaborative approach prevented unnecessary procedures and enabled safe reversion to rivaroxaban therapy. Previous studies have highlighted the importance of multimodality cardiac imaging in the assessment and management of cardiac masses in the hope of avoiding invasive cardiac biopsy [15].

As this case illustrates, even with advanced modalities such as CMR, diagnostic certainty is maximised through MDT interpretation [16]. Integrating multimodal imaging findings within a multidisciplinary framework ensures accurate diagnosis, prevents overtreatment, and aligns management with the patient’s clinical context.

## Conclusions

This case reinforces the importance of multimodality cardiac imaging and MDT review in the assessment of suspected cardiac masses. Such collaborative, integrated approaches are essential to ensure accurate diagnosis and avoid unnecessary or potentially harmful interventions.
